# Harnessing the abscopal effect: mechanistic insights and therapeutic strategies for systemic cancer immunotherapy

**DOI:** 10.3389/fimmu.2026.1871187

**Published:** 2026-07-30

**Authors:** Linxi Cao, Siqi Tong, Haihui Jiang

**Affiliations:** Department of Neurosurgery, Peking University Third Hospital, Peking University, Beijing, China

**Keywords:** abscopal effect, immunotherapy, radiotherapy, tumor immunology, tumor microenvironment

## Abstract

The abscopal effect, defined as the regression of non-irradiated tumors following localized radiotherapy (RT), represents one of the most intriguing manifestations of systemic antitumor immunity. Although traditionally considered a rare and unpredictable phenomenon, increasing evidence indicates that the abscopal effect is an immune-mediated process initiated by RT-induced immunogenic cell death. In addition to directly eliminating tumor cells, RT promotes the release of tumor-associated antigens and danger-associated molecular patterns, activates antigen-presenting cells, and primes tumor-specific T-cell responses, thereby triggering systemic immune surveillance. The development of immunotherapy, particularly immune checkpoint inhibitors (ICIs), has substantially enhanced the likelihood of inducing abscopal responses by sustaining antitumor immunity and overcoming tumor-induced immune suppression. As a result, the abscopal effect has evolved from an anecdotal clinical observation into a promising strategy for systemic cancer treatment. However, its clinical application remains limited by tumor heterogeneity, the immunosuppressive tumor microenvironment, uncertainties regarding optimal radiation dose, fractionation, and treatment sequencing, as well as the lack of reliable predictive biomarkers. In this review, we summarize the current understanding of the biological mechanisms underlying the abscopal effect, discuss its reciprocal interaction with immunotherapy, and evaluate recent advances in radio-immunotherapy. We also highlight the major challenges that impede clinical translation and discuss future strategies for biomarker development, treatment optimization, and precision radio-immunotherapy. Overall, this review provides a comprehensive overview of the current landscape of the abscopal effect and offers perspectives on its development as a predictable and clinically applicable therapeutic strategy.

## Introduction

Cancer remains one of the most formidable challenges to global public health, with its incidence and mortality continuing to rise despite substantial advances in diagnosis and treatment. According to the Global Burden of Disease (GBD) 2023 systematic analysis, the global cancer burden is expected to reach approximately 30.5 million new cases and 18.6 million cancer-related deaths annually by 2050 (1). Against this backdrop, radiotherapy (RT) continues to serve as a cornerstone of cancer management, with more than half of patients receiving RT during the course of their disease (2). Traditionally, the therapeutic efficacy of RT has been attributed primarily to localized DNA damage and the consequent induction of tumor cell death, leading to its long-standing classification as a local treatment modality (3, 4).

This conventional paradigm has been challenged by the recognition of the abscopal effect, a rare phenomenon in which localized RT induces regression of distant, non-irradiated tumor lesions (4). First described by Mole in 1953 (5), the abscopal effect remained largely anecdotal for decades because of its infrequent occurrence and the absence of a clear biological explanation. Consequently, it was long regarded as an unpredictable clinical curiosity rather than a reproducible therapeutic phenomenon.

The emergence of cancer immunology has reshaped this perspective. Increasing evidence demonstrates that RT not only eradicates tumor cells through direct cytotoxicity but also remodels the tumor-immune ecosystem by inducing immunogenic cell death, promoting the release of tumor-associated antigens and damage-associated molecular patterns (DAMPs), activating antigen-presenting cells, and priming tumor-specific T-cell responses (6, 7). In this context, RT has been recognized as an *in situ* vaccine capable of initiating systemic antitumor immunity beyond the irradiated field. More importantly, the remarkable clinical success of immunotherapy, particularly immune checkpoint inhibitors (ICIs), has increased both the incidence and reproducibility of abscopal responses, transforming the abscopal effect from an isolated clinical observation into a promising strategy for systemic cancer immunotherapy (8, 9).

Despite these encouraging advances, the clinical translation of the abscopal effect remains challenging. Objective abscopal responses are observed in only a small subset of patients and are influenced by multiple factors, including tumor immunogenicity, the immunosuppressive tumor microenvironment (TME), host immune competence, and treatment-related variables such as radiation dose, fractionation schedule, treatment sequencing, and the selection of combination therapies (10). Furthermore, the molecular determinants governing the initiation, amplification, and maintenance of systemic antitumor immunity remain incompletely understood, and reliable predictive biomarkers capable of identifying patients most likely to benefit are still lacking.

In this review, we provide a comprehensive and mechanistically integrated overview of the abscopal effect, with particular emphasis on the molecular and cellular mechanisms linking radiotherapy to systemic immune activation. We further summarize current clinical evidence supporting radioimmunotherapy combinations, discuss the major barriers limiting successful clinical translation, and highlight emerging therapeutic strategies aimed at enhancing the frequency and durability of abscopal responses. By integrating mechanistic insights with translational and clinical advances, this review seeks to provide a conceptual framework for harnessing the abscopal effect as a predictable and clinically actionable modality for systemic cancer control.

## The discovery and evolution of the abscopal effect

The abscopal effect, first described by Mole in 1953 (5), refers to the regression of distant, non-irradiated tumors following localized RT (11). For decades, this phenomenon was regarded as an exceedingly rare and unpredictable clinical observation, documented primarily through sporadic case reports (12, 13). Early evidence was most frequently reported in hematological malignancies, particularly lymphomas, which are characterized by a highly immunogenic microenvironment and abundant immune cell infiltration (14–16). Although these early observations hinted at an immune-mediated mechanism, the biological basis of the abscopal effect remained largely speculative because the interplay between RT and antitumor immunity was poorly understood.

With the rapid advancement of tumor immunology, the conceptual framework of the abscopal effect has undergone a fundamental transformation. Rather than acting solely as a localized cytotoxic treatment, RT is now recognized as a potent immunomodulatory intervention capable of inducing immunogenic cell death, enhancing antigen presentation, activating innate immune signaling, and priming systemic tumor-specific T-cell responses (17, 18). Consequently, the abscopal effect is no longer viewed as a stochastic event but as a biologically plausible consequence of RT-induced systemic immune activation (19–21).

This conceptual shift has been reinforced by parallel advances in radiation delivery and immunotherapy. Modern RT techniques, including stereotactic body radiotherapy (SBRT), intensity-modulated radiotherapy (IMRT), and image-guided radiotherapy (IGRT), have substantially enhanced the precision and biological efficacy of RT (22). More importantly, the clinical introduction of ICIs has increased both the frequency and durability of abscopal responses, providing compelling evidence that appropriate modulation of antitumor immunity can convert this once exceptional phenomenon into a reproducible therapeutic strategy.

**Figure 1:**
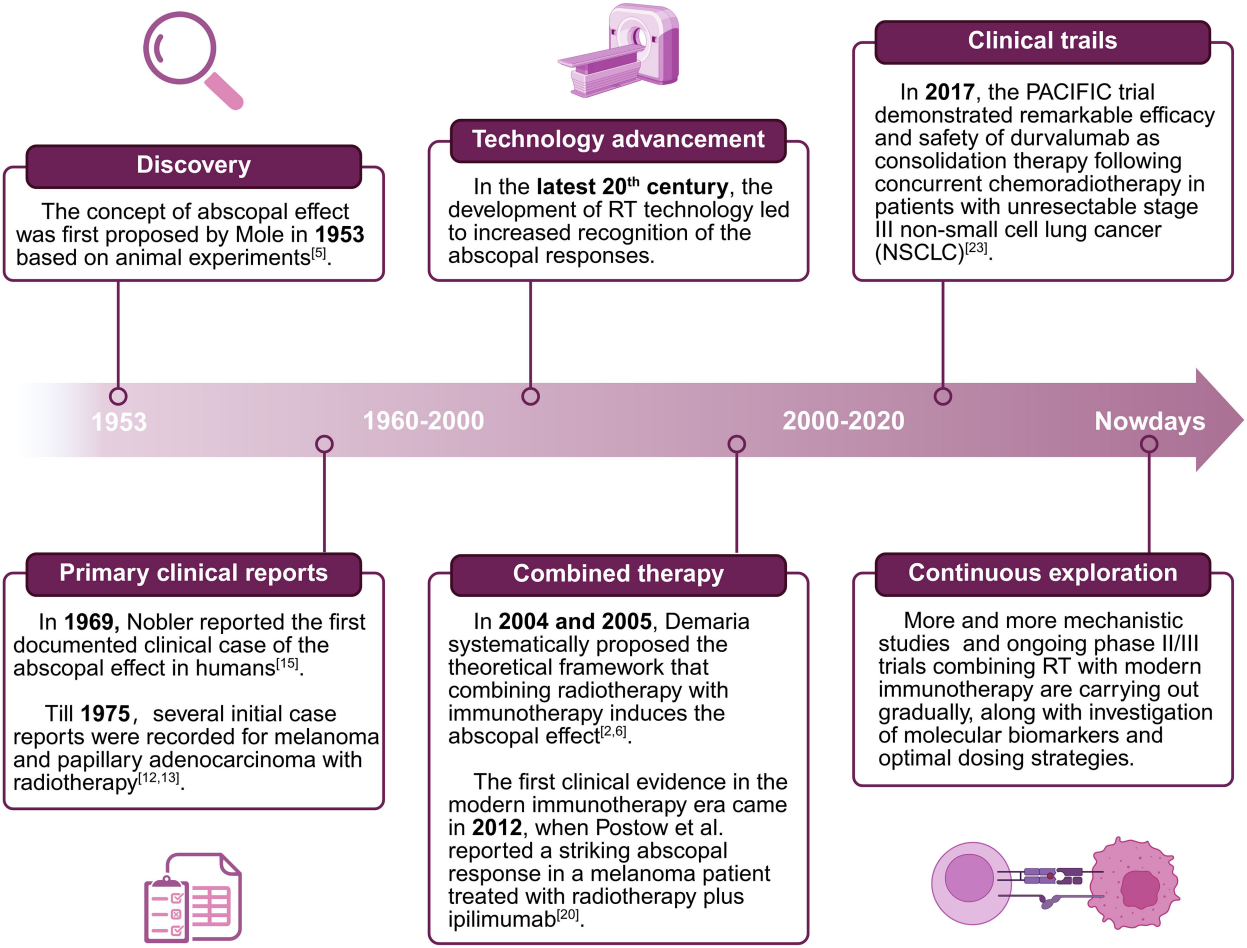
General timeline for the abscopal effect in RT. Key milestones include its initial description by Mole in 1953, early clinical reports prompted by the advanced technology (1960s–2000), recognition of its immunological basis, and the dramatic increase in incidence following the integration of RT with immunotherapy (2000–present). Ongoing studies focus on molecular biomarkers, optimal dosing, and large-scale clinical validation. Created with BioRender.com.

Nevertheless, the abscopal effect remains uncommon and highly heterogeneous across tumor types, individual patients, and treatment settings (11). This variability reflects the complex interactions among tumor intrinsic characteristics, the tumor microenvironment, host immune status, and treatment-related variables, including radiation dose, fractionation regimen, and treatment sequencing (23). A deeper mechanistic understanding of these determinants will therefore be essential for transforming the abscopal effect from an infrequent clinical observation into a predictable and clinically exploitable therapeutic modality (**Figure 1**).

## The biological mechanisms underlying the abscopal effect

**Figure 2:**
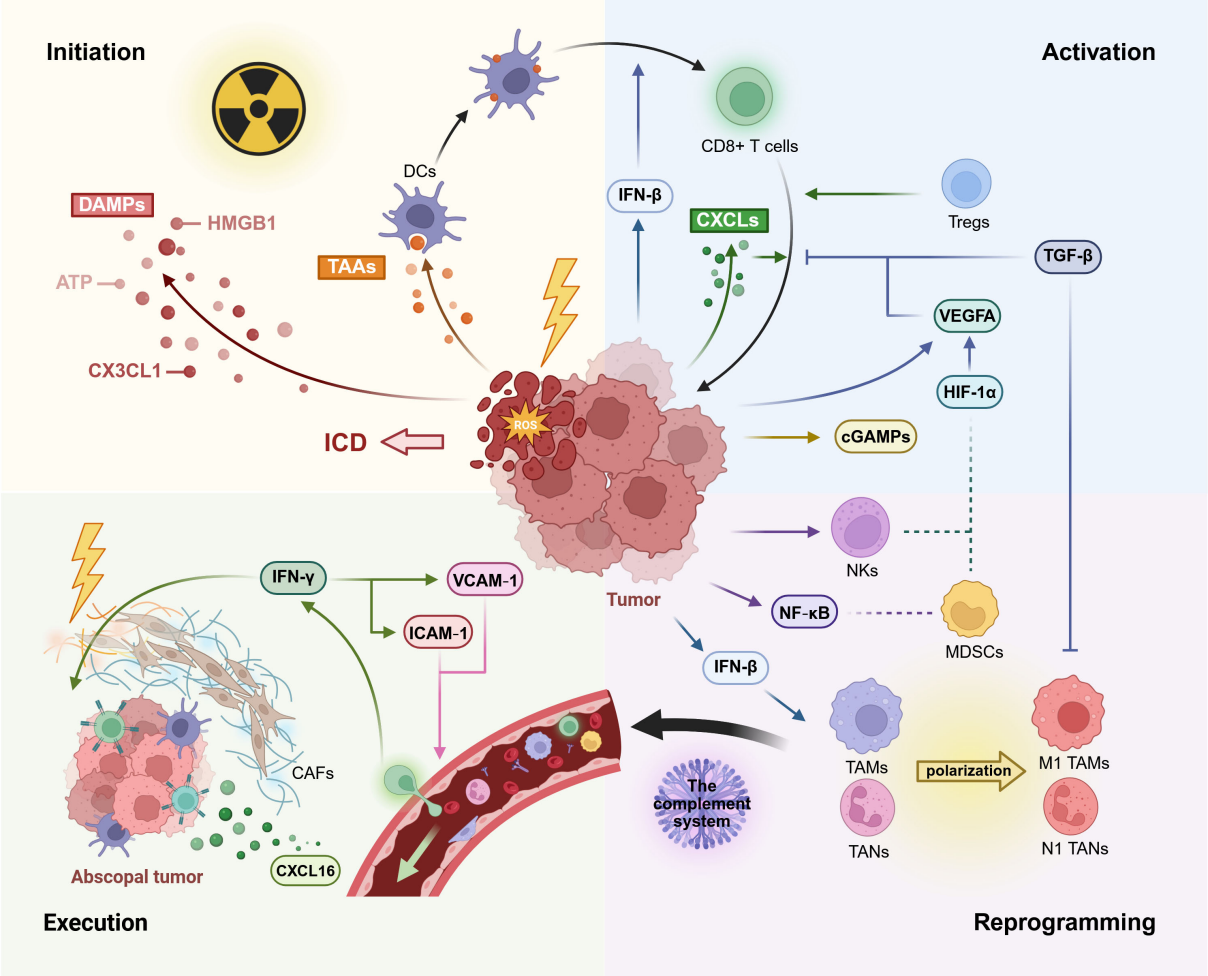
Schematic illustration of the four key phases of the abscopal effect. The four phases of the abscopal effect, including Initiation (antigen release and priming), Activation (cGAS–STING-mediated IFN-β and CXCLs production), Reprogramming (TME remodeling driven by TGF-β, HIF-1α and NF-κB), and Execution (systemic trafficking of effector CD8^+^ T cells and NK cells to distant abscopal tumors). Created with BioRender.com.

The abscopal effect is now recognized as the culmination of a coordinated immune cascade rather than an isolated consequence of localized irradiation (24). Although its molecular basis remains incompletely understood, accumulating evidence supports a multistep process that links local radiation-induced tumor damage to systemic immune surveillance (4). Mechanistically, this process can be divided into four interconnected phases: initiation, immune activation, tumor microenvironment reprogramming, and systemic immune execution (**Figure 2**).

### Initiation phase: tumor antigen release and immunogenic priming

The initiation of the abscopal effect depends on the conversion of irradiated tumors from passive targets of radiation into active sources of immune stimulation (25). This transformation is primarily driven by radiation-induced immunogenic cell death (ICD), a specialized form of cell death that simultaneously eliminates malignant cells and generates potent immunostimulatory signals (26). In addition to inducing irreversible DNA damage, RT increases tumor immunogenicity by enhancing neoantigen availability and upregulating major histocompatibility complex (MHC) expression, thereby improving antigen visibility to the immune system (27).

Unlike apoptotic cell death, ICD elicits immune activation through the coordinated release of damage-associated molecular patterns (DAMPs), including high-mobility group box 1 (HMGB1), adenosine triphosphate (ATP), and C-X3-C motif chemokine ligand 1 (CX3CL1), largely as a consequence of reactive oxygen species-mediated endoplasmic reticulum stress (28–32). These endogenous signals recruit dendritic cells (DCs) to the irradiated lesion, promote their maturation, and facilitate efficient uptake and cross-presentation of tumor-associated antigens to CD8^+^ T cells (28, 33). Consequently, the irradiated tumor functions as an *in situ* vaccine, initiating adaptive immune responses capable of extending beyond the radiation field.

HMGB1 and ATP remain the canonical mediators of radiation-induced ICD (34, 35). Nevertheless, emerging evidence suggests that additional regulators also influence immunogenic priming. For example, protein regulator of cytokinesis 1 (PRC1) has recently been implicated in modulating ICD through the wingless-related integration site/β-catenin (Wnt/β-catenin) signaling pathway, highlighting that radiation-induced immunogenicity is governed by a complex regulatory network rather than a limited set of danger signals alone (36).

### Activation phase: innate sensing and adaptive immune amplification

The release of tumor antigens alone is insufficient to generate systemic antitumor immunity. Productive abscopal responses require efficient innate immune sensing that converts local radiation-induced damage into adaptive immune activation. Among the pathways identified to date, the cyclic GMP–AMP synthase (cGAS)–stimulator of interferon genes (STING) axis represents the central molecular hub linking RT to immune activation.

**Figure 3:**
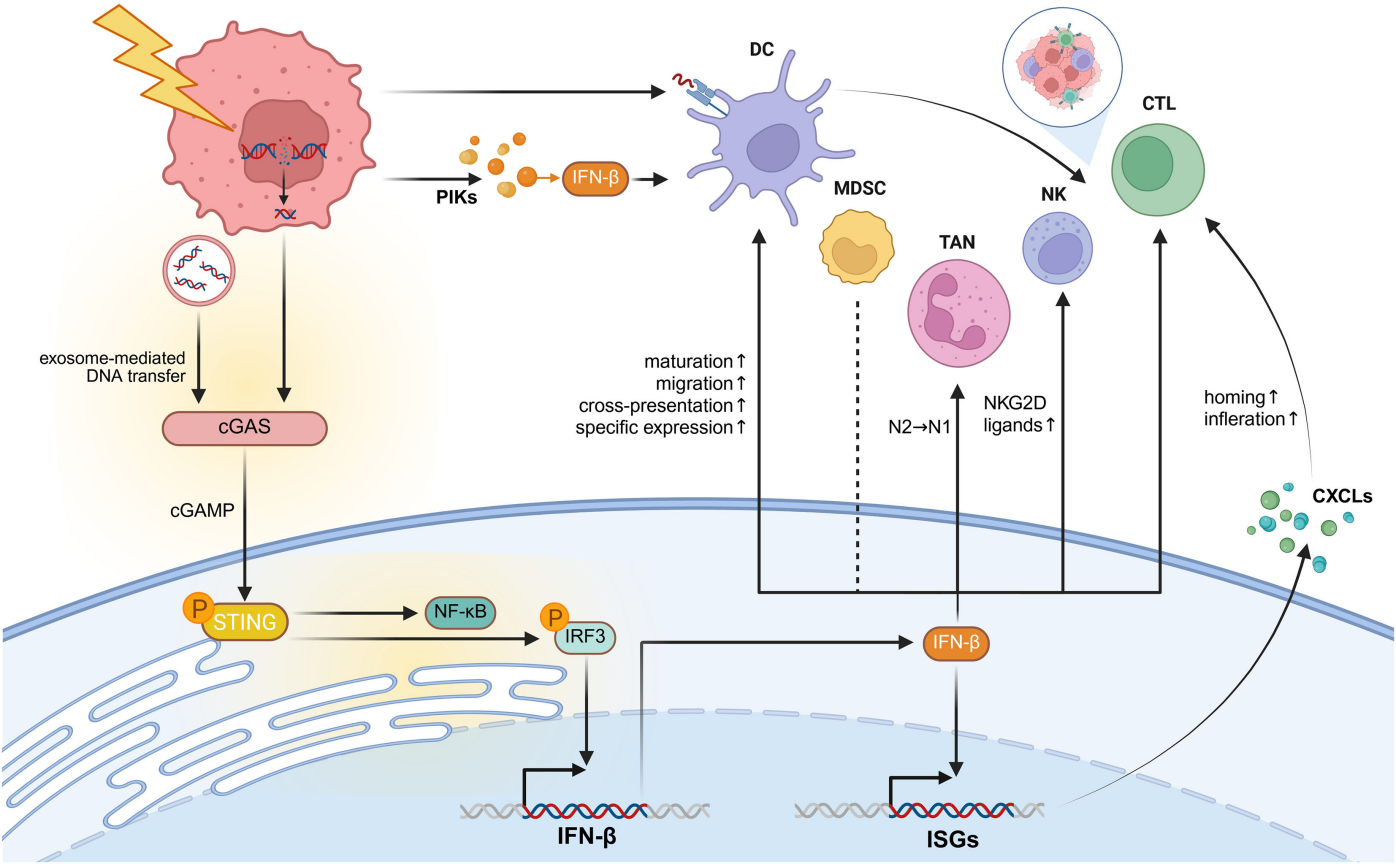
cGAS-STING pathway in RT-induced antitumor immunity. IR causes DNA damage, inducing the release of proinflammatory and micronuclei formation in tumor cells, activating cGAS to produce cGAMP. cGAMP activates STING, leading to TBK1-mediated phosphorylation of IRF3 and NF-κB, ultimately driving IFN-β production. IFN-β promotes CXCLs secretion, which enhances the tumor-homing capacity of both NK cells and T cells. It also activates immune cell, including DC maturation, migration, cross-presentation and specific expression, NKG2D ligand upregulation, and neutrophil polarization towards N1. Exosome-mediated DNA/cGAMP transfer further amplifies the response in non-irradiated cells, enabling systemic antitumor immunity, i.e., abscopal effect. Created with BioRender.com.

**Figure 4:**
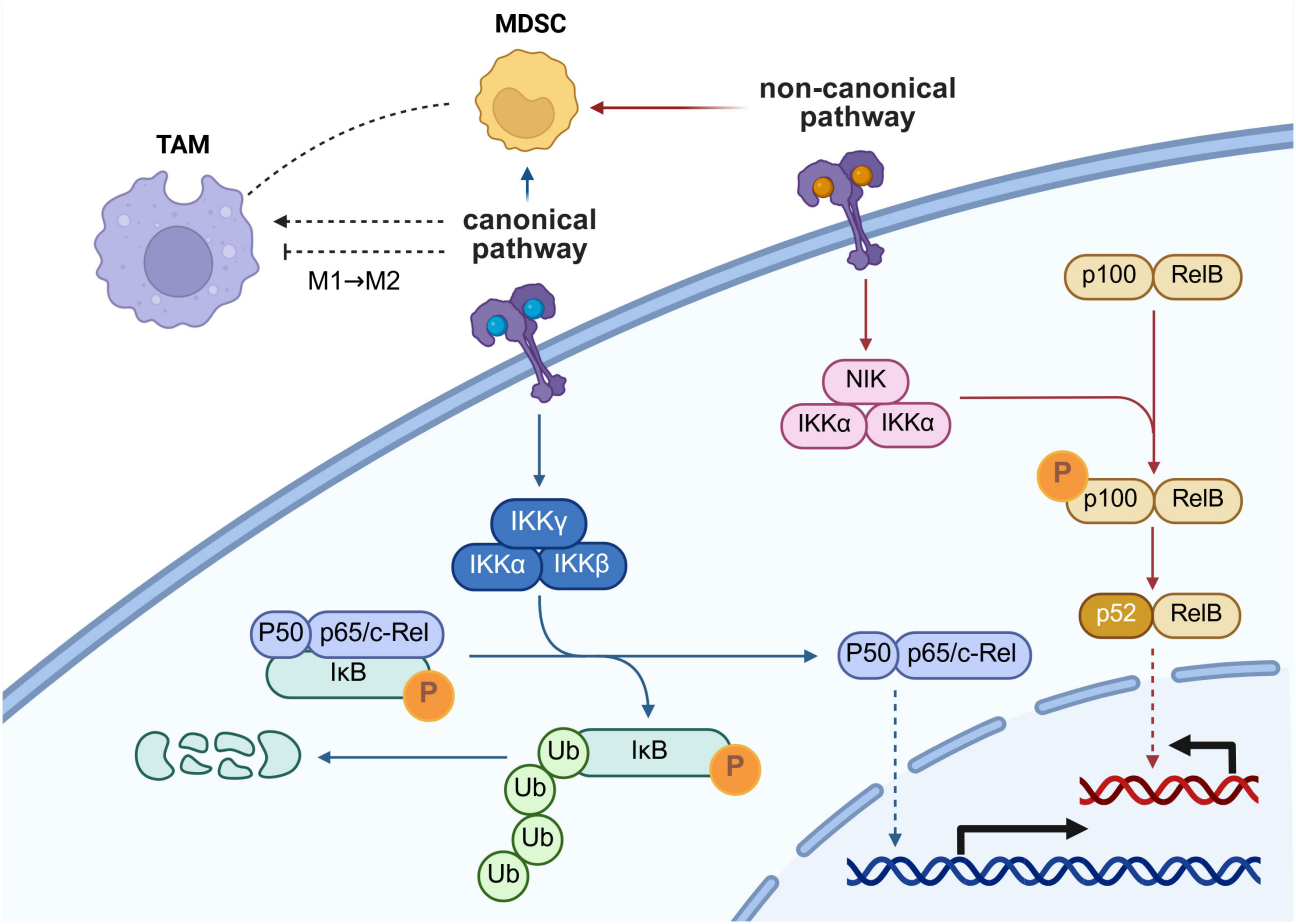
NF-κB signaling pathway in the tumor microenvironment following RT. NF-κB is activated through two distinct pathways: the canonical pathway, which generates p50/RELA and c-Rel dimers in an IκB-dependent manner, and the non-canonical pathway, which produces p52/RelB dimers via NIK/IKKα. In the context of RT, NF-κB acts as a central signaling hub that drives immunosuppression by promoting MDSC accumulation through the non-canonical pathway and exerting context-dependent effects on TAM polarization via the canonical pathway, thereby facilitating tumor immune evasion. Created with BioRender.com.

Radiation-induced DNA damage results in the accumulation of cytosolic DNA fragments and micronuclei, which are recognized by cGAS (37). Activated cGAS catalyzes the production of cyclic GMP–AMP (cGAMP), leading to STING activation and subsequent phosphorylation of TBK1 and interferon regulatory factor 3 (IRF3). This signaling cascade culminates in robust production of type I interferons, particularly interferon-β, together with nuclear factor kappa-light-chain-enhancer of activated B cells (NF-κB)-dependent inflammatory cytokines that establish a pro-inflammatory immune milieu (38–41) (**Figures 3**, **4**).

Type I interferons occupy a pivotal position within this cascade because they bridge innate immune sensing with adaptive immune priming. They promote DC maturation and migration to draining lymph nodes, enhance antigen cross-presentation, and support the expansion and functional differentiation of tumor-specific CD8^+^ T cells (42–45). Simultaneously, interferon-inducible chemokines, including C-X-C motif chemokine ligand 10 (CXCL10) and C-X-C motif chemokine ligand 10 (CXCL16), recruit activated lymphocytes into both irradiated and distant tumors, thereby amplifying systemic immune surveillance (46). In addition, intercellular transfer of cGAMP between tumor cells and neighboring stromal or immune cells further propagates STING signaling beyond the irradiated lesion, extending local immune activation into a systemic response (47).

**Figure 5:**
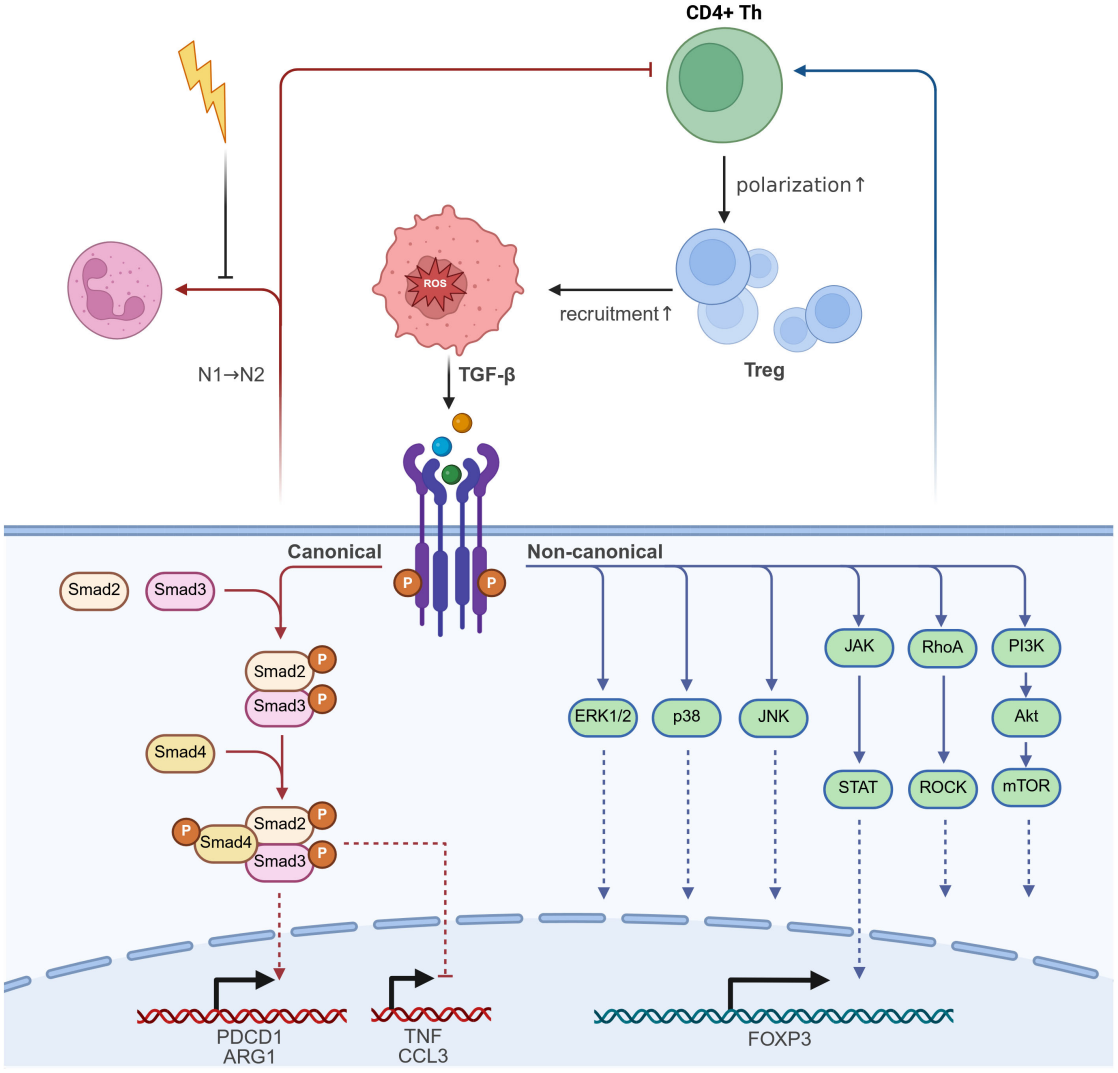
Canonical and non-canonical TGF-β signaling pathways in tumor immune regulation. IR induces ROS production in tumor cells, triggering TGF-β release. In the canonical pathway, TGF-β activates Smad2/3/4 complexes for long-term gene regulation. Through this way, TGF-β exerts mainly immunosuppressive effects by driving TANs toward the pro-tumor N2 phenotype, inducing Treg recruitment and upregulating PD-L1 to induce T-cell exhaustion. During this process, RT may transfer the favor of TGF-β to N1 polarization, leading to antitumor activity. Besides, there are several routes in the non-canonical pathway, which elicit more rapid responses.

**Figure 6:**
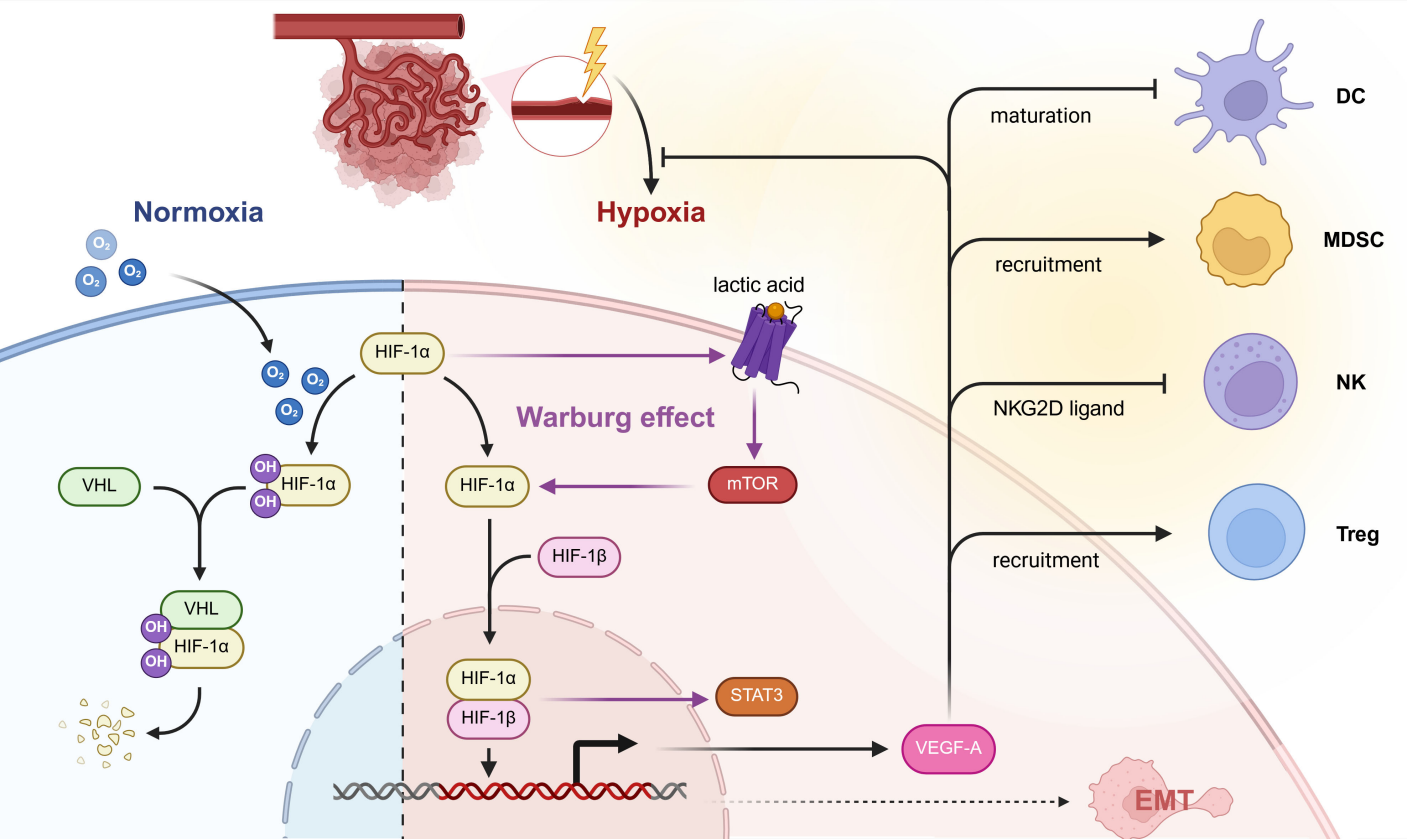
HIF-1α signaling pathway under hypoxic conditions induced by RT. Radiation-induced vascular damage exacerbates tumor hypoxia, stabilizing HIF-1α. This activates downstream effectors including VEGF-A, PD-L1, and STAT3, promoting MDSC and Treg recruitment, inhibiting DC maturation, and reducing NK cell cytotoxicity via MICA shedding. HIF-1α also mediates the Warburg effect, interacts with lactate/mTOR signaling to further enhance immunosuppression. HIF-1α also transcriptionally upregulates several critical Epithelial–Mesenchymal Transition (EMT) transcription factors, thereby directly promoting epithelial–mesenchymal transition and enhancing tumor cell invasion, metastasis, and therapeutic resistance. Created with BioRender.com.

The magnitude of this immune activation is, however, tightly constrained by intrinsic negative regulatory mechanisms. Trex1, a DNA exonuclease induced by high-dose irradiation, degrades cytosolic DNA and thereby limits cGAS activation, providing a mechanistic basis for the dose dependence of radiation-induced immunity (18). Meanwhile, radiation-associated hypoxia and oxidative stress activate immunosuppressive pathways dominated by hypoxia-inducible factor-1α (HIF-1α) and transforming growth factor-β (TGF-β), which impair DC function, promote regulatory T-cell differentiation, increase programmed death-ligand 1 (PD-L1) expression, and collectively restrain systemic immune activation (48–52) (**Figures 5**, **6**). Therefore, whether RT ultimately promotes an abscopal response depends largely on the dynamic balance between immune activation and compensatory immunosuppressive feedback.

### Reprogramming phase: tumor microenvironment remodeling

Even robust T-cell priming rarely translates into durable systemic tumor control unless accompanied by profound remodeling of the tumor microenvironment (TME). The immune composition and functional status of the TME ultimately determine whether activated effector cells can infiltrate tumors and exert sustained cytotoxic activity.

RT reshapes the TME through coordinated regulation of multiple innate immune populations. Type I interferon signaling promotes polarization of tumor-associated macrophages and neutrophils toward pro-inflammatory M1 and N1 phenotypes, whereas persistent TGF-β signaling favors immunosuppressive M2 and N2 polarization, thereby limiting antitumor immunity (53–55). Likewise, myeloid-derived suppressor cells (MDSCs) reinforce immune evasion through activation of NF-κB, HIF-1α, and Janus Kinase – Signal Transducer and Activator of Transcription 3 (JAK–STAT3) signaling pathways, although the magnitude of these effects varies substantially across tumor types and treatment settings (56, 57).

Natural killer (NK) cells provide an additional layer of early immune surveillance. RT enhances NK-cell recruitment and cytotoxicity by inducing stress-associated ligands on tumor cells and augmenting interferon signaling (58). However, hypoxia-induced shedding of ligands such as MICA may attenuate NK-cell function, illustrating the dynamic competition between immune stimulation and immune suppression within the irradiated TME (59).

Beyond cellular remodeling, complement signaling has recently emerged as another potential regulator of radiation-induced immunity (60, 61). Although complement activation has traditionally been associated with tumor progression, accumulating evidence suggests that selected complement components may facilitate DC activation, antigen presentation, and T-cell priming following RT (61–63). These observations underscore the context-dependent nature of complement signaling and suggest that its immunological functions extend beyond conventional inflammatory responses (63).

### Execution phase: systemic immune trafficking and distant tumor elimination

The defining hallmark of the abscopal effect is the capacity of locally primed immune cells to eradicate tumors outside the irradiated field. Following activation within draining lymph nodes, tumor-specific effector T cells undergo systemic expansion and subsequently migrate to distant metastatic lesions, where they execute antigen-specific cytotoxicity (64).

Successful immune trafficking requires coordinated remodeling of stromal, vascular, and chemokine networks. RT can modify the phenotype of cancer-associated fibroblasts (CAFs), alleviating stromal exclusion and facilitating lymphocyte infiltration. Conversely, excessive or inappropriate irradiation may induce a senescence-associated secretory phenotype that promotes tumor progression and immune suppression, highlighting the dual role of CAFs in radiation responses (65–69). At the vascular level, interferon-γ induces endothelial adhesion molecules, including intercellular adhesion molecule 1 (ICAM-1) and vascular cell adhesion molecule 1 (VCAM-1), while chemokines such as CXCL16 establish gradients that direct activated T cells toward metastatic lesions (70–73).

Long-range immune communication is further reinforced through extracellular vesicles and intercellular transfer of immune mediators, extending radiation-induced immune signaling beyond the primary treatment site (74–76). Nevertheless, systemic antitumor immunity remains susceptible to inhibitory mediators, including plasminogen activator inhibitor-1 (PAI-1) and other radiation-induced inflammatory factors that dampen immune effector function (66).

Taken together, the abscopal effect should not be regarded as the consequence of a single molecular pathway but rather as the emergent outcome of a highly integrated immune network. Effective systemic tumor regression requires successful completion of each stage of the immune cascade—from immunogenic tumor cell death and innate immune sensing to adaptive immune activation, TME remodeling, and immune cell trafficking. Failure at any point along this continuum may prevent the establishment of durable abscopal immunity, providing a mechanistic explanation for the rarity and heterogeneity of this phenomenon in clinical practice.

## The interaction between the abscopal effect and immunotherapy

Advances in understanding the immune basis of the abscopal effect have transformed its clinical significance from an infrequent observation to a rational therapeutic strategy (77, 78). Rather than acting as independent modalities, radiotherapy and immunotherapy function synergistically, with radiation initiating antitumor immune responses that can be amplified and sustained by immunotherapeutic interventions (10). This reciprocal interaction has established the biological rationale for combining these approaches and provides the framework for optimizing systemic antitumor immunity (10).

### Immunotherapy potentiates the abscopal effect

The clinical success in enhancing the abscopal effect has been achieved through ICIs. Although RT efficiently initiates tumor-specific immune responses, these responses are frequently attenuated by compensatory immune inhibitory pathways activated within the tumor microenvironment. ICIs overcome these adaptive resistance mechanisms, allowing radiation-primed lymphocytes to expand, persist, and mediate systemic tumor eradication (79, 80).

Among currently available checkpoint pathways, the PD-1/PD-L1 axis represents the dominant mechanism of adaptive immune escape in many solid tumors (81). Engagement of PD-1 expressed on activated T cells with PD-L1 on tumor or immune cells suppresses T-cell proliferation, cytokine production, and cytotoxic activity while simultaneously reinforcing an immunosuppressive microenvironment (81). Blocking this pathway preserves the function of radiation-induced tumor-reactive T cells and prevents their functional exhaustion (79). Consequently, combined RT and PD-1/PD-L1 inhibition consistently enhances CD8^+^ T-cell infiltration, interferon-γ production, and regression of both irradiated and distant tumors in preclinical models (80, 82, 83). The encouraging clinical efficacy observed across multiple malignancies has consequently established anti-PD-1 and anti-PD-L1 antibodies as the therapeutic backbone of contemporary radioimmunotherapy strategies (79, 84–86).

Notably, recent studies have broadened the understanding of PD-1 blockade beyond simple reinvigoration of exhausted T cells. PD-1 inhibition also facilitates the expansion of newly primed T-cell clones generated following RT-induced antigen release, thereby increasing the diversity of tumor-reactive lymphocytes participating in systemic immune surveillance (87, 88). These findings suggest that checkpoint inhibition not only restores immune competence but also actively reshapes the evolving antitumor immune repertoire.

cytotoxic T-lymphocyte-associated protein 4 (CTLA-4) blockade complements PD-1 inhibition by targeting an earlier stage of the immune response. By competing with CD28 for binding to CD80 and CD86, CTLA-4 negatively regulates T-cell priming within secondary lymphoid organs (89, 90). Inhibition of CTLA-4 therefore enhances activation of naïve T cells while simultaneously reducing regulatory T-cell-mediated immune suppression (89, 91). Although CTLA-4 inhibitors are associated with greater immune-related toxicity and generally display lower efficacy as monotherapy than PD-1/PD-L1 inhibitors (92, 93), dual checkpoint blockade provides complementary regulation of both T-cell priming and effector function, offering a strong biological rationale for maximizing RT-induced systemic immunity (89).

Beyond checkpoint blockade, emerging immunotherapeutic strategies further reinforce distinct stages of the radiation-induced immune cascade (94, 95). Adoptive cell therapies, particularly chimeric antigen receptor (CAR)-T cells, provide large numbers of tumor-specific cytotoxic lymphocytes capable of exploiting the antigen-rich inflammatory environment generated by RT (94). Recent advances, including PD-1-deficient CAR-T cells and cytokine-secreting constructs, have substantially improved resistance to the immunosuppressive tumor microenvironment and may facilitate durable systemic immune propagation following irradiation (94, 96). Likewise, dendritic-cell vaccines enhance antigen presentation and long-term immune memory, thereby strengthening the initial immune priming induced by RT (95, 97–99). Oncolytic viruses provide additional synergy by combining direct tumor lysis with local immune activation, further increasing antigen release and inflammatory signaling (100). Early clinical studies integrating intratumoral viral therapy with RT have demonstrated encouraging systemic responses, highlighting the therapeutic potential of simultaneously targeting multiple components of the cancer–immunity cycle (101, 102).

Collectively, these approaches illustrate a common biological principle: while RT initiates systemic immune activation, immunotherapy sustains and amplifies this response by preventing immune exhaustion and overcoming suppressive mechanisms. Successful induction of the abscopal effect therefore depends not merely on antigen release but on continuous reinforcement of antitumor immunity throughout the entire immune cascade.

### The abscopal effect facilitates immunotherapy

Despite the transformative success of cancer immunotherapy, durable clinical benefit remains limited to a subset of patients. Primary resistance, acquired immune escape, and treatment-related toxicities continue to restrict therapeutic efficacy across many solid tumors (103–106). These limitations largely arise from insufficient immune priming, poor lymphocyte infiltration, stromal exclusion, and profoundly immunosuppressive tumor microenvironments, all of which constrain effective antitumor immunity (107).

The biological processes underlying the abscopal effect directly address many of these barriers. Through induction of immunogenic cell death, RT generates abundant tumor antigens and danger-associated molecular signals that promote dendritic-cell maturation and antigen cross-presentation (108). Simultaneously, radiation-induced inflammatory cytokines and chemokines facilitate recruitment of effector lymphocytes into previously immune-excluded tumors (80). RT also enhances major histocompatibility complex class I expression, remodels tumor vasculature, and partially disrupts stromal barriers, collectively improving immune-cell trafficking and tumor recognition (80, 108, 109). These coordinated changes convert immunologically “cold” tumors into inflamed, immune-responsive lesions, thereby creating a permissive microenvironment for immunotherapy (80).

Beyond local immune modulation, RT initiates systemic tumor-specific T-cell responses that provide the mechanistic foundation upon which immunotherapy can achieve durable systemic tumor control (110, 111). Clinical studies have demonstrated that stereotactic body radiotherapy administered before or concurrently with immune checkpoint blockade significantly improves objective response rates compared with immunotherapy alone (112). Similar synergistic benefits have been observed in multiple emerging combination strategies targeting complementary immunosuppressive pathways, further supporting the concept that effective immunotherapy requires coordinated activation of the entire cancer–immunity cycle rather than isolated modulation of individual checkpoints (113).

Overall, the abscopal effect has evolved from a rare clinical observation into a conceptual framework that reshapes modern cancer immunotherapy (114). It establishes RT as an active immune-modulating intervention rather than merely a cytotoxic modality, emphasizes the necessity of integrating local and systemic immune activation, and provides a biological rationale for rational combination therapies (80, 111). Furthermore, because successful abscopal responses reflect productive systemic immune activation, biomarkers associated with this phenomenon may facilitate patient stratification, optimize treatment sequencing, and improve prediction of immunotherapy responsiveness (114).

## Clinical implications and future perspectives

The recognition of the abscopal effect has fundamentally reshaped the role of radiotherapy in modern oncology (77, 78). Rather than serving solely as a local cytotoxic modality, radiotherapy is now increasingly viewed as an immune-modulating intervention capable of initiating systemic antitumor responses (4, 115). Nevertheless, despite substantial advances in elucidating its molecular basis and encouraging results from radio-immunotherapy trials, the abscopal effect remains an infrequent and largely unpredictable clinical event (4). Most reported cases continue to derive from case reports, retrospective analyses, or early-phase clinical studies, and the incidence of reproducible systemic responses varies considerably among tumor types and treatment settings (116). Consequently, the central challenge has shifted from demonstrating that the abscopal effect exists to understanding how it can be induced consistently and translated into a reliable therapeutic strategy (115).

### Precision prediction of abscopal responses

A major obstacle to clinical implementation is the absence of robust biomarkers capable of identifying patients who are likely to develop systemic immune responses following RT (114). Numerous candidate biomarkers have been investigated (114), including post-radiotherapy absolute lymphocyte count (ALC) (117), tumor-infiltrating lymphocytes (TILs) (118), T-cell receptor (TCR) repertoire and clonality (119), circulating tumor DNA (ctDNA) (120), PD-L1 expression (121–123), tumor mutational burden (TMB) (121–123), ICAM-1 (124), and transcriptomic signatures such as gene expression profiling (GEP) and the Tumor Immune Dysfunction and Exclusion (TIDE) score (122, 125). Collectively, these biomarkers capture different aspects of systemic immunity, tumor immunogenicity, or immune escape (114, 125, 126). However, none alone adequately reflects the dynamic and multistep immune processes required for successful abscopal responses, and most supporting evidence remains exploratory or retrospective (78, 114) (**Table 1**).

**Table 1:** Biomarkers of the abscopal effect.

Biomarker	Cutoff value	Endpoint/key results	Sensitivity	Specificity	Cancer type	Phase	Evidence level	Reference
Post-radiotherapy absolute lymphocyte count (pre-RT ALC)	Median split; 0.56 x 10^3 cells/mL; above median vs below median	Abscopal response in nonirradiated lesions: 34.2% above median vs 3.9% below median; P<0.0001; PFS: 12.3 vs 6.0 months; P = 0.0004; multivariable PFS HR 0.544; 95% CI 0.363-0.815; P = 0.003; OS: 27.4 vs 15.7 months; P = 0.005; multivariable OS association P = 0.026	/	/	Multiple cancer types; NSCLC; SCLC; head and neck cancer; renal cell carcinoma; others	Retrospective analysis of 3 phase 1/2 trials; n=153	C	Absolute Lymphocyte Count Predicts Abscopal Responses and Outcomes in Patients Receiving Combined Immunotherapy and Radiation Therapy: Analysis of 3 Phase 1/2 Trials (117)
Pre-radiotherapy absolute lymphocyte count (pre-RT ALC)	Median split; 1.3 x 10^3 cells/mL; above median vs below median	Abscopal response in nonirradiated lesions: 30.3% above median vs 7.8% below median; P = 0.0004; not retained as a significant multivariable predictor; PFS: 7.8 vs 6.6 months; P = 0.21; OS: 19.2 vs 22.8 months; P = 0.82	/	/	Multiple cancer types; NSCLC; SCLC; head and neck cancer; renal cell carcinoma; others	Retrospective analysis of 3 phase 1/2 trials; n=153	C	Absolute Lymphocyte Count Predicts Abscopal Responses and Outcomes in Patients Receiving Combined Immunotherapy and Radiation Therapy: Analysis of 3 Phase 1/2 Trials (117)
Peripheral blood TCR repertoire Shannon diversity change	Change from baseline; unchanged defined as <1% deviation from baseline; decreased/stable vs increased	Response/progression monitoring; decreasing or stable diversity in the best responders; increased diversity at progression in 4/5 patients with initial stable disease and available progression samples; baseline diversity was not associated with best response or PD-L1 status; formal diagnostic performance not reported	/	/	Stage IV NSCLC	Exploratory translational analysis in a phase 2 trial; ComIT-1; n=15; PBMC samples n=46	D	Dynamic changes in the T cell receptor repertoire during treatment with radiotherapy combined with an immune checkpoint inhibitor (119)
New high-abundance TCR clones after radiotherapy	New clones among the top 100 most frequent TCRs after treatment; network analysis excluded TCRs with frequency <0.001	Radiotherapy as a personalized cancer vaccine; new high-abundance TCRs at radiotherapy were observed in 3/4 PR; 1/4 SD; 0/2 PD; no formal diagnostic performance reported	/	/	Stage IV NSCLC	Exploratory translational analysis in a phase 2 trial; ComIT-1; n=15; PBMC samples n=46	D	Dynamic changes in the T cell receptor repertoire during treatment with radiotherapy combined with an immune checkpoint inhibitor (119)
Fecal gut microbiome alpha diversity (inverse Simpson score)	High >11.63; intermediate 7.46-11.63; low <7.46	Anti-PD-1 response; PFS; responders had significantly higher alpha diversity; P<0.01; median PFS undefined for high diversity; 232 days for intermediate diversity; 188 days for low diversity; high vs intermediate diversity HR 3.60; 95% CI 1.02-12.74; high vs low diversity HR 3.57; 95% CI 1.02-12.52	/	/	Metastatic melanoma	Prospective cohort; human fecal samples n=43; additional translational mouse fecal microbiota transplant experiments	C	Gut microbiome modulates response to anti-PD-1 immunotherapy in melanoma patients (126)
Fecal crOTU community type 1 (Ruminococcaceae/Faecalibacterium-enriched type)	Unsupervised crOTU community type; type 1 vs type 2	Anti-PD-1 response; type 1: R = 11/11; NR = 0/11; type 2: R = 19/32; NR = 13/32; Fisher’s exact test showed association with response	11/30 36.7%; using type 1 to predict response; calculated from reported counts	13/13 100%; using type 1 to predict response; calculated from reported counts	Metastatic melanoma	Prospective cohort; human fecal samples n=43; additional translational mouse fecal microbiota transplant experiments	C	Gut microbiome modulates response to anti-PD-1 immunotherapy in melanoma patients (126)
Faecalibacterium genus abundance	High vs low abundance; universal numeric cutoff not reported	PFS; high abundance median PFS undefined vs low abundance 242 days; HR 2.92; 95% CI 1.08-7.89; multivariable HR 2.95; 95% CI 1.31-7.29; P = 0.03; positively correlated with tumor CD8+ T-cell density; R2 = 0.42; P<0.01	/	/	Metastatic melanoma	Prospective cohort; human fecal samples n=43; additional translational mouse fecal microbiota transplant experiments	C	Gut microbiome modulates response to anti-PD-1 immunotherapy in melanoma patients (126)
Bacteroidales abundance	High vs low abundance; universal numeric cutoff not reported	Nonresponse tendency; PFS; high Bacteroidales median PFS 188 days vs low Bacteroidales 393 days; HR 0.39; 95% CI 0.15-1.03; enriched in nonresponders; associated with Tregs/MDSCs and a blunted cytokine response	/	/	Metastatic melanoma	Prospective cohort; human fecal samples n=43; additional translational mouse fecal microbiota transplant experiments	C	Gut microbiome modulates response to anti-PD-1 immunotherapy in melanoma patients (126)
ICAM-1 expression/ICAM-1-targeted PET or NIRF signal	High vs low; based on Dye-alphaICAM-1/Fab fluorescence intensity or 64Cu-NOTA-alphaICAM-1/Fab PET uptake; no universal numeric cutoff	Abscopal response/nonirradiated tumor growth inhibition; in 4T1, ICAM-1-high nonirradiated tumors grew more slowly than ICAM-1-low tumors; n=6 vs n=8; P<0.01; CT26 showed the same predictive pattern; P<0.05; 64Cu PET signal negatively correlated with relative nonirradiated tumor volume; r=-0.875; P<0.001	/	/	Mouse 4T1 breast cancer; CT26 colon cancer; validation in human irradiated tumor tissues	Basic laboratory and animal experiments; human irradiated tumor tissue validation; nonclinical predictive performance study	D	ICAM-1 orchestrates the abscopal effect of tumor radiotherapy (124)
ICAM-1 expression on tumor-infiltrating CD8+ T cells	High vs low; numeric cutoff not reported	Mechanism/prediction of abscopal response; ICAM-1 expression on CD8+ cells was significantly higher in responder nonirradiated tumors; ICAM-1 blockade almost fully reversed RT-induced nonirradiated tumor inhibition; CD8 blockade showed a similar pattern; associated with CD8+ T-cell activation and infiltration	/	/	Mouse 4T1 breast cancer; CT26 colon cancer; validation in human irradiated tumor tissues	Basic laboratory and animal experiments; human irradiated tumor tissue validation; nonclinical predictive performance study	D	ICAM-1 orchestrates the abscopal effect of tumor radiotherapy (124)
Tumor mutational burden (TMB; FoundationOne CDx)	>=10 mutations/Mb defined as high TMB; secondary nivolumab monotherapy analysis used >=13 mutations/Mb	PFS; ORR; in the high-TMB population, nivolumab plus ipilimumab vs chemotherapy: 1-year PFS 42.6% vs 13.2%; median PFS 7.2 vs 5.5 months; HR 0.58; 97.5% CI 0.41-0.81; P<0.001; ORR 45.3% vs 26.9%; high-TMB evaluable population 444/1004	/	/	Stage IV or recurrent NSCLC	Phase 3 randomized open-label trial; CheckMate 227 part 1	A	Nivolumab plus Ipilimumab in Lung Cancer with a High Tumor Mutational Burden (121)
PD-L1 expression	>=1% vs <1%	Benefit stratification in the high-TMB population; nivolumab plus ipilimumab benefit was consistent in the PD-L1 >=1% subgroup with PFS HR 0.62; 95% CI 0.44-0.88; and in the PD-L1 <1% subgroup with HR 0.48; 95% CI 0.27-0.85; TMB and PD-L1 were not correlated; high-TMB benefit was largely independent of PD-L1	/	/	Stage IV or recurrent NSCLC	Phase 3 randomized open-label trial; CheckMate 227 part 1	A	Nivolumab plus Ipilimumab in Lung Cancer with a High Tumor Mutational Burden (121)
Tumor mutational burden (TMB; WES nonsynonymous mutations/exome)	Youden cutoffs: pan-tumor 102.5 mutations/exome; HNSCC 86; melanoma 191.5; exploratory pan-tumor cutoff >=123; another reported threshold >=175	BOR/ORR; PFS; AUROC pan-tumor 0.740; HNSCC 0.617; melanoma 0.602; PFS TMBhi vs TMBlo: pan-tumor 115 vs 59 days; HR 0.48; HNSCC 64 vs 64 days; HR 0.70; melanoma 502 vs 85 days; HR 0.48	/	/	22 advanced solid tumor types; HNSCC; melanoma; other KEYNOTE cohort tumors	Retrospective biomarker analysis of 4 KEYNOTE clinical trials; >300 samples; TCGA validation analysis	B	Pan-tumor genomic biomarkers for PD-1 checkpoint blockade-based immunotherapy (122)
T cell-inflamed gene expression profile (18-gene GEP)	GEP score >= -0.318 defined as GEPhi; 18 genes include CCL5; CD27; CD274/PD-L1; CD276; CD8A; CMKLR1; CXCL9; CXCR6; HLA-DQA1; HLA-DRB1; HLA-E; IDO1; LAG3; NKG7; PDCD1LG2; PSMB10; STAT1; TIGIT	BOR/ORR; PFS; AUROC pan-tumor 0.782; HNSCC 0.768; melanoma 0.638; PFS GEPhi vs GEPlo: pan-tumor 96 vs 57 days; HR 0.54; HNSCC 103 vs 57 days; HR 0.45; melanoma 418 vs 90 days; HR 0.73	/	/	22 advanced solid tumor types; HNSCC; melanoma; other KEYNOTE cohort tumors	Retrospective biomarker analysis of 4 KEYNOTE clinical trials; >300 samples; TCGA validation analysis	B	Pan-tumor genomic biomarkers for PD-1 checkpoint blockade-based immunotherapy (122)
Combined TMBhi + GEPhi status	TMB at or above cohort-specific Youden cutoffs; GEP >= -0.318	BOR/ORR; PFS; GEPhi/TMBhi ORR 37-57%; GEPhi/TMBlo ORR 12-35%; GEPlo/TMBhi ORR 11-42%; GEPlo/TMBlo ORR 0-9%; PFS TMBhi/GEPhi vs TMBlo/GEPlo: pan-tumor 189 vs 59 days; HR 0.43; HNSCC 110 vs 62 days; HR 0.51; melanoma 504 vs 123 days; HR 0.63	/	/	22 advanced solid tumor types; HNSCC; melanoma; other KEYNOTE cohort tumors	Retrospective biomarker analysis of 4 KEYNOTE clinical trials; >300 samples; TCGA validation analysis	B	Pan-tumor genomic biomarkers for PD-1 checkpoint blockade-based immunotherapy (122)
Microsatellite instability-high (MSI-H; mSINGS WES)	mSINGS MSI score >20% unstable microsatellite loci; PCR confirmation	Pembrolizumab response; 2 MSI-H tumors were identified and both were responders; gastric carcinoma n=1; biliary tract carcinoma n=1; sample size too small to estimate diagnostic performance	/	/	22 advanced solid tumor types; gastric carcinoma; biliary tract carcinoma	Retrospective biomarker analysis of 4 KEYNOTE clinical trials; >300 samples; TCGA validation analysis	B	Pan-tumor genomic biomarkers for PD-1 checkpoint blockade-based immunotherapy (122)
PD-L1 IHC expression combined with TMB	PD-L1 positivity varied by KEYNOTE cohort and tumor type; HNSCC approximately CPS or modified proportion score >=1; melanoma MEL score >=2 with membrane staining in tumor and immune cells	Joint stratification for pembrolizumab response; TMB plus PD-L1 results were comparable with TMB plus GEP results; GEP correlated with PD-L1 at r=0.49-0.53; P<0.001; TMB had low or no correlation with PD-L1	/	/	22 advanced solid tumor types; HNSCC; melanoma; other KEYNOTE cohort tumors	Retrospective biomarker analysis of 4 KEYNOTE clinical trials; >300 samples; TCGA validation analysis	B	Pan-tumor genomic biomarkers for PD-1 checkpoint blockade-based immunotherapy (122)
PD-L1 tumor proportion score (22C3 IHC)	>=50% membranous staining in neoplastic tumor cells; additional groups <1% and 1-49%; cutoff selected from the training cohort by ROC analysis	ORR; PFS; OS; training cohort >=50% ORR 36.6%; validation cohort >=50% ORR 45.2%; 95% CI 33.5-57.3; all patients with >=50% had median PFS 6.3 months; median OS not reached; overall ORR 19.4%; screened prevalence of >=50% was 23.2%	/	/	Advanced NSCLC	Phase 1 clinical trial; KEYNOTE-001; training cohort n=182; validation cohort n=313	B	Pembrolizumab for the Treatment of Non-Small-Cell Lung Cancer (123)
PD-L1 score (Ventana SP263)	>=10% membranous staining in tumor cells	RECIST ORR in nonirradiated lesions; 7/20 patients had PD-L1 >=10%; 6/7 85% responded systemically; the other 3 responders had PD-L1 <1% or 1%; overall ORR in nonirradiated lesions was 9/20 45%; 3 CR + 6 PR	6/9 66.7%; using PD-L1 >=10% to predict RECIST response in nonirradiated lesions; calculated from reported counts	10/11 90.9%; using PD-L1 >=10% to predict RECIST response in nonirradiated lesions; calculated from reported counts	Metastatic or locally advanced inoperable melanoma	Open-label single-arm phase 2 clinical trial; n=20	C	Phase 2 Trial of Nivolumab Combined With Stereotactic Body Radiation Therapy in Patients With Metastatic or Locally Advanced Inoperable Melanoma (120)
Circulating tumor DNA (ctDNA; mutant BRAF/NRAS)	Detected vs not detected; decline after treatment or initial increase followed by decline; no fixed numeric cutoff	Dynamic monitoring of response/progression; ctDNA was detected in 8/20 patients; clinical responses and progression corresponded to ctDNA changes in 7/8 patients; 4 patients had an initial ctDNA increase after nivolumab followed by decline after SBRT; 3 had PR/CR and 1 had SD; exploratory marker for selecting patients who may need SBRT	/	/	Metastatic or locally advanced inoperable melanoma	Open-label single-arm phase 2 clinical trial; n=20	C	Phase 2 Trial of Nivolumab Combined With Stereotactic Body Radiation Therapy in Patients With Metastatic or Locally Advanced Inoperable Melanoma (120)
Local complete response in the irradiated lesion (abscopal/local response)	Complete response in the irradiated lesion	Systemic complete response in nonirradiated lesions; all patients with CR in nonirradiated lesions also had local CR in the irradiated lesion; proposed as a surrogate marker; diagnostic performance not validated	/	/	Metastatic or locally advanced inoperable melanoma	Open-label single-arm phase 2 clinical trial; n=20	C	Phase 2 Trial of Nivolumab Combined With Stereotactic Body Radiation Therapy in Patients With Metastatic or Locally Advanced Inoperable Melanoma (120)
TIDE score (T cell dysfunction and exclusion transcriptome signature)	CTL-high if CD8A; CD8B; GZMA; GZMB; and PRF1 were all above the dataset average; TIDE threshold varied across ROC analyses; survival Kaplan-Meier used top (>1) vs bottom (<1) TIDE prediction scores	ICB response; OS; PFS; TIDE predicted first-line anti-PD1 or anti-CTLA4 melanoma outcomes more accurately than PD-L1 level; mutation load; and IFNG response; higher TIDE score was associated with worse ICB response and worse OS/PFS; exact AUC values were shown in figures but not printed in the extracted main text	/	/	Melanoma as the main immunotherapy validation setting; transcriptomic modeling across 33 cancer types	Computational biomarker development and multi-cohort validation using public datasets; accompanied by experimental functional validation	C	Signatures of T cell dysfunction and exclusion predict cancer immunotherapy response (125)
SERPINB9 expression	Top half vs bottom half expression; IFN-gamma-induced expression	ICB resistance; OS; PFS; SERPINB9 was lower in responders than nonresponders; high SERPINB9 was associated with worse OS/PFS in anti-CTLA4 cohorts; CRISPR knockout increased sensitivity to T cell-mediated killing; overexpression increased resistance	/	/	Melanoma; multi-cancer expression cohorts	Computational biomarker development and multi-cohort validation using public datasets; accompanied by experimental functional validation	C	Signatures of T cell dysfunction and exclusion predict cancer immunotherapy response (125)
CTL marker set (CD8A; CD8B; GZMA; GZMB; PRF1)	All 5 markers above the dataset average defined CTL-high; all other tumors defined CTL-low	Branching variable in TIDE; CTL-high tumors used the dysfunction signature; CTL-low tumors used the exclusion signature; CTL level alone was insufficient for accurate ICB response prediction	/	/	Melanoma as the main immunotherapy validation setting; transcriptomic modeling across 33 cancer types	Computational biomarker development and multi-cohort validation using public datasets; accompanied by experimental functional validation	C	Signatures of T cell dysfunction and exclusion predict cancer immunotherapy response (125)
TIL score in distant/untreated tumors (stromal; intratumor; invasive margin)	No fixed diagnostic cutoff; post-treatment increase vs baseline	Abscopal effect; recurrence/metastasis; AbsCryo stromal TIL 2.8 +/- 1.1% vs baseline 1.0 +/- 0%; P = 0.015; invasive margin TIL 50 +/- 12.2% vs baseline 31 +/- 4.9%; P = 0.02; postprocedure TIL increase was associated with prevention of recurrence; P = 0.02; cryoablation recurrence/metastasis 0/5 0%; resection recurrence/metastasis 2/5 40%	/	/	4T1-12B triple-negative breast cancer mouse model	Basic laboratory/animal experiment; bilateral breast cancer mouse pilot study; resection n=5; cryoablation n=5	D	Tumor-Infiltrating Lymphocytes (TILs) as a Biomarker of Abscopal Effect of Cryoablation in Breast Cancer: A Pilot Study (118)
CD8a+ T cells and granzyme B in distant tumors	Normalized immunofluorescence signal change; no fixed diagnostic cutoff	Abscopal CTL response; AbsCryo CD8a 15.7 +/- 12.1 vs baseline 5.2 +/- 4.7; P = 0.020; granzyme B 4.8 +/- 3.6 vs baseline 2.4 +/- 0.9; P = 0.048; indicates increased activated CTLs in distant tumors	/	/	4T1-12B triple-negative breast cancer mouse model	Basic laboratory/animal experiment; bilateral breast cancer mouse pilot study; resection n=5; cryoablation n=5	D	Tumor-Infiltrating Lymphocytes (TILs) as a Biomarker of Abscopal Effect of Cryoablation in Breast Cancer: A Pilot Study (118)

ALC, absolute lymphocyte count; RT, radiotherapy; TCR, T cell receptor; crOTU, clustered ribosomal operational taxonomic unit; WES, whole-exome sequencing; TMB, tumor mutational burden; MSI-H, microsatellite instability-high; PD-L1, programmed death-ligand 1; CPS, combined positive score; TPS, tumor proportion score; IHC, immunohistochemistry; GEP, gene expression profile; TIDE, Tumor Immune Dysfunction and Exclusion; CTL, cytotoxic T lymphocyte; TIL, tumor-infiltrating lymphocyte; ctDNA, circulating tumor DNA; ICAM-1, intercellular adhesion molecule-1; NIRF, near-infrared fluorescence; PET, positron emission tomography; ICI, immune checkpoint inhibitor; SBRT, stereotactic body radiation therapy; PBMC, peripheral blood mononuclear cell; TCGA, The Cancer Genome Atlas; FMT, fecal microbiota transplantation; HR, hazard ratio; CI, confidence interval; AUROC, area under receiver operating characteristic curve; ROC, receiver operating characteristic; BOR, best overall response; ORR, objective response rate; PFS, progression-free survival; OS, overall survival; RECIST, Response Evaluation Criteria in Solid Tumors; NSCLC, non-small cell lung cancer; SCLC, small cell lung cancer; HNSCC, head and neck squamous cell carcinoma; RCT, randomized controlled trial; R, responder; NR, non-responder;/, not available.

Among currently available biomarkers, preservation of post-radiotherapy lymphocyte counts has generated the most consistent clinical evidence (114, 117). A pooled analysis of three phase I/II clinical trials demonstrated that patients maintaining higher post-treatment ALC experienced markedly higher abscopal response rates and superior survival outcomes than those with severe radiation-induced lymphopenia (117). Because lymphocytes constitute the principal mediators of systemic antitumor immunity, these observations underscore the importance of preserving immune competence during radiotherapy (117). Nevertheless, biomarkers reflecting tumor immunogenicity, including PD-L1 expression and TMB, have yielded inconsistent predictive performance across different malignancies and immunotherapeutic regimens (114, 121–123, 127). These findings suggest that the abscopal effect is unlikely to be predicted by any single molecular marker. Instead, future biomarker development will likely evolve toward integrated prediction models that combine longitudinal peripheral immune monitoring, tumor genomic characteristics, spatial features of the tumor microenvironment, and circulating biomarkers through multi-omics approaches and computational modeling (78, 114).

### Optimizing radio-immunotherapy as an integrated therapeutic strategy

Patient selection alone is insufficient to maximize systemic immune responses. The efficacy of the abscopal effect also depends critically on rational optimization of treatment design (78). Increasing evidence indicates that successful induction of systemic immunity is governed by multiple interacting variables, including radiation dose and fractionation, irradiation target selection, treatment sequencing, lymphoid tissue preservation, and the choice of immunotherapeutic agents (78, 128, 129). Rather than acting independently, these parameters collectively determine the magnitude, quality, and persistence of radiation-induced immune activation (78).

The temporal relationship between RT and ICI administration is a critical determinant of therapeutic efficacy, as it governs the kinetics of radiation-induced immune activation (130, 131). Current evidence generally supports concurrent or closely sequential ICI administration, preferably within 14 days after RT, to maximize treatment efficacy (116, 130, 132–134). However, computational modeling by Sakai et al. suggests that the optimal treatment schedule is context-dependent: effective priming and expansion of naïve T cells require tight temporal coupling between RT and ICI, whereas reinvigoration of exhausted T cells may be achieved over a broader therapeutic window (131).

RT dose and fractionation represent a second critical determinant of therapeutic efficacy, reflecting a non-monotonic relationship between radiation dose and immune activation. Preclinical studies consistently identify hypofractionated RT, particularly stereotactic body radiation therapy (SBRT) delivering 10–13 Gy per fraction, as the regimen that best balances antitumor immune stimulation with the avoidance of immunosuppressive effects when combined with ICIs (10, 18, 135–138). This observation is increasingly supported by emerging clinical evidence (139, 140). Mechanistically, excessively high single doses (>15 Gy) promote abnormal expansion of splenic regulatory T cells, whereas fractional doses exceeding 12–18 Gy induce Trex1 expression, resulting in degradation of cytosolic DNA, attenuation of cGAS-STING signaling, and impaired CD8^+^ T-cell activation (18, 138). By contrast, conventional fractionation (2 Gy×10) preserves both peripheral and tumor-infiltrating CD8^+^ T cells, reduces MDSC infiltration, and enhances type I interferon production and major histocompatibility complex (MHC)-I expression when combined with anti-PD-1 therapy, leading to superior control of both primary and distant tumors in preclinical models (135). Nevertheless, the optimal dose-fractionation strategy remains dependent on tumor type and immune context (137).

Selection of the irradiated lesion and the number of treated sites constitute additional determinants of therapeutic efficacy that extend beyond technical considerations. Optimal site selection should take into account immunological accessibility, the capacity to support systemic T-cell priming and trafficking, and the local immunosuppressive microenvironment (10, 141). Because tumor heterogeneity limits the effectiveness of single-site irradiation by restricting the breadth of tumor-associated antigens available for immune priming while allowing site-specific immunosuppressive niches to persist (142), multisite irradiation is being explored as a strategy to overcome these limitations (143–148). By broadening antigen exposure and alleviating immunosuppression across multiple tumor sites, multisite RT promotes a more diverse polyclonal antitumor T-cell response and may enhance systemic therapeutic efficacy (143).

Accumulating evidence suggests that no universal treatment paradigm is likely to exist (78, 149). Instead, the optimal treatment strategies will probably vary according to tumor immunogenicity, metastatic burden, host immune status, and the specific mechanisms of immune resistance operating within individual tumors (78, 114). Consequently, future clinical studies should move beyond evaluating isolated treatment variables toward developing adaptive and mechanism-based therapeutic strategies (78, 111). Integration of serial immune monitoring, functional imaging, and translayer molecular analyses into prospective clinical trials may enable dynamic adjustment of treatment protocols and facilitate individualized radio-immunotherapy (78, 114).

### Tumor heterogeneity of the abscopal effect

The probability and magnitude of the abscopal effect vary substantially across tumor types, reflecting differences in immunogenicity, TME composition, and the immunological pathways engaged by RT (150–155). Representative abscopal response rates are summarized in **Table 2**.

**Table 2:** Clinical trials of the abscopal effect in the past decade.

Patient amount	Main results	Major toxic reactions	Start date	Completion date	The irradiated area of tumor regression	Occurrence rate of the abscopal effect	Reference
28 patients	ORR 75.0% (95% CI 59-91); DCR 100% (95% CI 100-100); median PFS 16.8 months (95% CI 11.5-NR); median OS 25.4 months (95% CI 13.3-NR); multiomics suggested cryoablation increased tumor immunogenicity and dendritic-cell activation; lenvatinib transitioned endothelial cells toward inflamed venules and promoted lymphocyte influx	All 28 patients had TRAEs; common TRAEs fatigue 68%; hypertension 65%; hypoalbuminemia 57%; hypothyroidism 57%; grade 3 TRAEs 10/28 36%; grade 4 hyperbilirubinemia 1/28 4% led discontinuation	2021-08-24	2024-03-02	Non-cryoablated target lesions; intrahepatic and/or extrahepatic lesions outside ablated area	ARR 21/28 75.0%; PR 19/28 67.9%; CR 2/28 7.1%; ACR/DCR 28/28 100%	Cryoablation plus sintilimab and lenvatinib in advanced or metastatic intrahepatic cholangiocarcinoma: a phase 2 trial (150)
37 enrolled; 30 ITT/safety after exclusions; 26 efficacy-evaluable	Median follow-up 5.97 months; median PFS 3.50 months (95% CI 2.73-4.23); ORR 23.08%; DCR 65.38%; OS immature; 6 PR and 11 SD among 26 evaluable patients; target lesions had PR 7 and SD 18; abscopal effects 6/26 23.08%; 4 of 6 abscopal-effect patients had PR; abscopal effect associated with PR; NLR <5 associated with lower distant metastasis/death risk	17 AEs; six grade 3/4; no grade 5; main AEs hypothyroidism; reactive cutaneous capillary endothelial proliferation; thrombocytopenia; skin reactions; fever; two grade 4 events in same patient were thrombocytopenia and leukopenia; no RT dose reductions	2022-09	/	Unirradiated areas; mostly lung or mediastinum in reported abscopal-effect patients	ARR 6/26 23.08%; PR 6/26 23.08%; CR 0/26 0%	Hypofractionated radiotherapy combined with a PD-1 inhibitor, granulocyte macrophage colony stimulating factor, and thymosin-alpha1 in advanced metastatic solid tumors: a multicenter Phase II clinical trial (151)
69 enrolled; 63 evaluable	ITT ORR 12/69 17% with CR 1/69 1.4% and PR 11/69 16%; ITT DCR 38/69 55%; per-protocol ORR 19%; per-protocol DCR 60%; irradiated lesion ORR 29%; irradiated lesion DCR 85%; nonirradiated metastases ORR 12%; median PFS 5.6 months (95% CI 2.9-7.1); median OS 20 months (95% CI 17-NR); median TTR 2.8 months; median DOR 14 months	Grade 1–2 rash 13/69 19%; fatigue 12/69 17%; pneumonitis outside irradiated area 10/69 14%; endocrine events 4/69 5.8%; grade 3–4 TRAEs 18/69 26%; discontinuation for grade 3–4 AEs 5/69 7.2%; SBRT toxicity grade 1–2 only; no toxic deaths	2017-07-14	2019-03-04	Nonirradiated metastases	ARR 12% (nonirradiated metastases ORR); PR/; CR/	Nivolumab in Combination with Stereotactic Body Radiotherapy in Pretreated Patients with Metastatic Renal Cell Carcinoma. Results of the Phase II NIVES Study (128)
52 enrolled; 51 safety/efficacy analyzed	Overall confirmed irRC ORR 15/51 29.4%; clinical benefit 47/51 92.2%; IWG ORR 18/51 35.3%; IWG clinical benefit 45/51 88.2%; G100–20 mg cohort ORR 33.3%; overall G100 abscopal tumor reduction 24/37 64.9%; confirmed abscopal irRC response 6/37 16.2%; abscopal clinical benefit 27/37 73.0%; PFS 7.9 months for G100–20 mg; 9.1 months for G100–10 mg; 11.1 months for G100–10 mg+pembrolizumab	All patients had at least one AE; mostly grade 1 or 2; grade >=3 uncommon with G100 alone and higher with pembrolizumab; one grade 3 leukocytosis possibly related to G100–20 mg; pembrolizumab-related grade 3 hyponatremia/adrenal insufficiency/hypocalcemia/colitis in one patient with DLT colitis; no grade 5; no G100-related serious AEs	2016-02-03	2019-08-01	Non-treated distal tumor sites; outside radiation/injection field	ARR 6/37 16.2% confirmed abscopal irRC response; PR 6/37 16.2% irPR; CR 0/37 0% irCR; abscopal tumor reduction 24/37 64.9%; abscopal clinical benefit 27/37 73.0%	Phase 1/2 study of intratumoral G100 (TLR4 agonist) with or without pembrolizumab in follicular lymphoma (152)
Cohort A n=18; cohort B n=10 in full analysis set	Primary ORR in unirradiated lesions; cohort A ORR 2/18 11% (90% CI 4-29) and DCR 7/18 39%; cohort B ORR 0/10 0% and DCR 0%; median PFS cohort A 4.1 months (95% CI 2.1-6.1); cohort B 2.0 months (95% CI 1.2-3.7); 5 cohort A patients had SD >=24 weeks; all 23 tumor samples were PD-L1 negative	TRAEs cohort A 10/18 56%; cohort B 4/11 36%; common events hypothyroidism 33% in A and 18% in B; hyperthyroidism 28%; rash 22%; stomatitis/malaise/fever 11%; one grade 3 AST increase in cohort B; no grade >=3 in cohort A; no treatment-related deaths	2017-06-22	2018-11-30	Unirradiated target lesions; clinical benefit included breast and liver target lesions	Cohort A: ARR 2/18 11%; PR 2/18 11%; CR 0/18 0%; Cohort B: ARR 0/10 0%; PR 0/10 0%; CR 0/10 0%	Phase Ib/II study of nivolumab combined with palliative radiation therapy for bone metastasis in patients with HER2-negative metastatic breast cancer (153)
31 enrolled; 23 completed proton therapy/evaluable for completed-treatment analysis	ORR in target lesion outside radiation field used to assess abscopal effect; ITT ORR 7/31 22.6% with 1 CR and 6 PR; median OS 8.4 months (95% CI 2.5-14.3); median PFS 2.4 months (95% CI 0.6-4.2); among 23 patients completing proton therapy, ORR 7/23 30.4%; median OS 11.1 months; median PFS 3.7 months	Grade >=3 AEs 6/31 19.4%; anemia n=1; constipation n=1; electrolyte imbalance n=2; hyperglycemia n=1; pneumonia n=1	2018-04-11	2021-06-30	Target lesion(s) outside the radiation field	ARR 7/31 22.6% out-field response; PR 6/31 19.4%; CR 1/31 3.2%; proton-completed subgroup: ARR 7/23 30.4%; PR 6/23 26.1%; CR 1/23 4.3%	Phase II Trial of Combined Durvalumab Plus Tremelimumab with Proton Therapy for Recurrent or Metastatic Head and Neck Squamous Cell Carcinoma (156)
49 enrolled; 45 efficacy-evaluable	Median follow-up 12.8 months; median PFS 6.93 months (95% CI 4.60-9.26); median OS 12.8 months (95% CI 10.06-15.54); ITT ORR 20/49 40.8%; DCR 37/49 75.5%; CBR 26/49 53.1%; CR 4/49 8.2%; PR 16/49 32.6%; SD 17/49 34.7%; ARR 17/49 34.7%; ACR 34/49 69.4%; efficacy-evaluable ORR 20/45 44.4%; DCR 37/45 82.2%; ARR 17/45 37.8%; ACR 34/45 75.5%; PD-L1 CPS>=1 associated with higher ORR 66.67% vs 26.32% p=0.036	All patients had TRAEs; grade >=3 TRAEs 31/49 63.3%; common any-grade events myelosuppression; weight loss; fatigue; grade >=3 leukopenia 15/49 30.6%; lymphopenia 9/49 18.4%; thrombocytopenia 7/49 14.3%; positive fecal occult blood 5/49 10.2%; radiation pneumonia 13/49 26.5% with grade >=3 2/49 4.1%; radiation esophagitis 10/49 20.4% with grade >=3 3/49 6.1%; no treatment-related deaths	2018-11-19	/	Unirradiated lesions/oligometastases; included lung and lymph-node metastatic sites	ARR 17/49 34.7%; ACR 34/49 69.4%; PR/; CR/	Radiotherapy plus camrelizumab and irinotecan for oligometastatic esophageal squamous cell carcinoma patients after first-line immunotherapy plus chemotherapy failure: An open-label, single-arm, phase II trial (154)
55 enrolled; 51 safety population; 49 efficacy-evaluable	Median follow-up 32.1 months; ORR 18/49 36.7% (90% CI 25.3-49.5; 95% CI 23.4-51.7) meeting primary endpoint; DCR 33/49 67.3%; abscopal CR 5/49 10.2%; abscopal PR 10/49 20.4%; ASR 15/49 30.6% (95% CI 18.3-45.4); median PFS 5.9 months (95% CI 2.5-9.3); median OS 18.4 months (95% CI 9.7-27.1); 12/24/36-month OS 75.5%/56.1%/43.7%; SBRT increased Tfh and NK cells in unirradiated lesions; IL-2R/IL-21 associated with prognosis	Any-grade TRAEs 44/51 86.3%; grade 3 TRAEs 6/51 11.8%; no grade 4–5 TRAEs; common TRAEs fatigue 37.3%; fever 29.4%; ostealgia 17.6%; rash 11.8%; grade 3 immune-related AEs 3/51 5.9%; discontinuation of sintilimab in 5 patients due pneumonitis/myocarditis	2019-09-30	2022-08-31	Out-of-field/nonirradiated target lesions	ARR/ASR 15/49 30.6%; PR 10/49 20.4%; CR 5/49 10.2%	Sintilimab in combination with stereotactic body radiotherapy and granulocyte-macrophage colony-stimulating factor in metastatic non-small cell lung cancer: The multicenter SWORD phase 2 trial (157)
19 safety population; 14 efficacy-evaluable; 13 ICI-relapsed/refractory evaluable patients plus one ICI-naive PR patient	In 13 post-ICI-failure patients, ORR 3/13 23%; DCR 7/13 54% with 3 PR and 4 SD; responding tumors included NSCLC 2/2 with PR+SD; HNSCC 2/3 with PR+SD; Hodgkin lymphoma 2/2 PR after 12 or 16 prior lines; one Hodgkin lymphoma PR patient showed marked systemic abscopal PET-CT response; PR+SD patients had higher baseline GEF-H1 immune-activation scores and greater post-treatment DC activation/T-cell clonal expansion; in vitro IR then plinabulin induced strongest DC maturation and T-cell proliferation 51.0% vs plinabulin 22.1% vs DC alone 1.5%	All 19 had TEAEs; TRAEs 18/19 94.7%; grade 3/4 TRAEs 12/19 64.2%; common TRAEs hypertension 11/19 57.9% with grade 3/4 in 6; nausea 10/19 52.6%; ALT elevation 5/19 26.3%; AST elevation 4/19 21.1%; vomiting 4/19 21.1%; most other grade 3/4 TRAEs occurred in <15%	2021-04-14	2025-02-27	Unirradiated target lesions/systemic nonirradiated lesions; systemic PET-CT response in Hodgkin lymphoma	ARR/ORR 3/13 23% in unirradiated target lesions of post-ICI-failure patients; PR 3/13 23%; CR 0/13 0%; DCR 7/13 54%	Plinabulin following radiation enhances dendritic cell maturation and checkpoint inhibitor retreatment of relapsed/refractory cancers (155)

ARR, abscopal response rate; ACR, abscopal clinical response; ASR, abscopal survival rate; CR, complete response; PR, partial response; SD, stable disease; PD, progressive disease; ORR, objective response rate; DCR, disease control rate; CBR, clinical benefit response; DOR, duration of response; TTR, time to response; irRC, immune-related Response Criteria; IWG, immune working group; ITT, intention-to-treat population; PP, per-protocol population; PFS, progression-free survival; OS, overall survival; NR, not reached; CI, confidence interval; AE, adverse event; TRAE, treatment-related adverse event; DLT, dose-limiting toxicity; SBRT, stereotactic body radiation therapy; ICI, immune checkpoint inhibitor; NLR, neutrophil-to-lymphocyte ratio; PD-L1, programmed death-ligand 1; CPS, combined positive score; DC, dendritic cell; Tfh, follicular helper T cell; NK, natural killer cell;/, not available.

Melanoma and renal cell carcinoma (RCC) demonstrate the highest reported rates of abscopal responses, consistent with their inherently immunogenic phenotypes. In melanoma, Postow et al. provided the first clinical evidence linking the abscopal effect to systemic immune activation by showing that RT combined with ipilimumab induced New York esophageal squamous cell carcinoma 1 (NY-ESO-1)-specific CD4^+^ T cell expansion together with concomitant antibody responses (20). In renal cell carcinoma (RCC), abscopal responses have been consistently observed following the combination of ICIs and SBRT, likely facilitated by abundant baseline CD8^+^ T cell infiltration that lowers the threshold for RT-induced systemic immune activation (128, 158).

Non-small cell lung cancer (NSCLC) represents an intermediate immunogenic phenotype. RT-induced activation of the cGAS-STING pathway promotes type I interferon production, DC cross-priming, and CD8^+^ T cell trafficking to distant lesions. At the same time, interferon gamma (IFN-γ) signaling induces PD-L1 expression through the Janus Kinase (JAK), Signal Transducer and Activator of Transcription (STAT), and Interferon Regulatory Factor (IRF) pathway, establishing a compensatory immune checkpoint that can be effectively targeted by PD-1/PD-L1 blockade (159). Consistent with this mechanism, the phase II SWORD trial combining RT, GM-CSF, and ICIs achieved an abscopal response rate of 30.6%, accompanied by increased infiltration of Tfh and NK cells in non-irradiated lesions (157).

### Balancing efficacy with immune-related toxicity

As radio-immunotherapy becomes increasingly integrated into clinical practice, maintaining an appropriate balance between therapeutic efficacy and treatment-related toxicity will become equally important (111, 129). Current evidence indicates that combining radiotherapy with immune checkpoint inhibitors generally demonstrates an acceptable safety profile (111, 160). However, simultaneous activation of inflammatory pathways by both modalities inevitably increases the risk of overlapping toxicities (111, 128, 129).

Radiation-associated toxicities, including pneumonitis and esophagitis, may be potentiated during combination therapy, while immune-related adverse events such as dermatitis, colitis, hepatitis, endocrinopathies, and hematologic suppression also require careful surveillance (104, 105, 128, 129). Toxicity profiles further differ according to immunotherapeutic agents. CTLA-4 blockade is consistently associated with a higher incidence of severe immune-related adverse events, particularly colitis and hypophysitis (156, 161), whereas SBRT-based combination regimens generally remain well tolerated, with most adverse events limited to grade 1–2 and manageable through standard clinical interventions (128, 160). These observations emphasize that future optimization should focus not only on maximizing immune activation but also on preserving treatment tolerability through individualized risk assessment, multidisciplinary management, and early toxicity intervention (111, 129).

### Caution: negative trials on the abscopal effect

Despite compelling preclinical evidence supporting RT-induced systemic immune activation (162–165), several randomized clinical trials have failed to demonstrate a consistent clinical benefit (162, 166–168). In the phase II CHEckpoint Inhibition in Combination With an Immunoboost of External Beam Radiotherapy in Solid Tumors (CHEERS) trial, the addition of subablative multisite SBRT to ICIs failed to enhance abscopal responses in patients with advanced solid tumors (168). Similarly, in microsatellite stable (MSS) colorectal cancer, RT combined with PD-L1 and CTLA-4 blockade induced out-of-field tumor regression in only a single patient and was associated with a 42% incidence of grade 3–4 toxicities (166). Likewise, the addition of SBRT to dual immune checkpoint blockade failed to improve efficacy in Merkel cell carcinoma (167), and the MD Anderson Cancer Center (MDACC) trial reported comparable objective response rates (ORR) between combination therapy and ICIs alone in metastatic NSCLC (22% vs. 25%) (164).

Several biological mechanisms may account for these heterogeneous clinical outcomes. Low tumor immunogenicity, exemplified by MSS tumors with a low mutational burden, provides an unfavorable substrate for RT-induced systemic immune activation (166, 169). RT-induced lymphopenia may further compromise systemic antitumor immunity, highlighting the importance of preserving circulating lymphocytes to maximize the likelihood of an abscopal response (117, 170). In addition, persistent immunosuppressive networks within the irradiated TME may constrain effective *in situ* tumor vaccination despite successful T cell priming (117). Finally, irradiation of a single lesion is unlikely to overcome the antigenic and immunological heterogeneity characteristic of metastatic disease (165).

Nevertheless, negative findings from individual trials should not be interpreted as evidence against the biological validity of the abscopal effect. A pooled analysis of the Pembrolizumab After SBRT Versus Pembrolizumab Alone in Advanced NSCLC (PEMBRO-RT) and MDACC trials demonstrated a significantly higher abscopal response rate (41.7% vs. 19.7%) together with prolonged overall survival (19.2 vs. 8.7 months) in patients receiving combined RT and immunotherapy, underscoring that appropriate patient selection, radiation parameters, and trial design remain critical determinants of therapeutic success (171).

### Future directions: transforming the abscopal effect into predictable clinical benefit

Future progress in abscopal research will depend on bridging mechanistic insights with translational and clinical investigation (78, 111). Large multicenter prospective trials incorporating standardized radiotherapy protocols, harmonized definitions of abscopal responses, and comprehensive translational analyses will be essential to establish reproducible clinical evidence (78, 111, 129). Equally important will be the incorporation of longitudinal biospecimen collection, immune profiling, single-cell and spatial multi-omics technologies, and artificial intelligence-assisted predictive modeling to identify dynamic response signatures and guide personalized treatment strategies (111, 113, 114).

Beyond technological advances, a conceptual shift in how the abscopal effect is viewed may also be required. Rather than regarding it as a rare biological curiosity or an isolated therapeutic endpoint, the abscopal effect should be considered a measurable indicator of successful systemic immune activation. Within this framework, the occurrence of an abscopal response reflects effective initiation and maintenance of the cancer-immunity cycle by radiotherapy (78, 111, 113). Future therapeutic development should therefore prioritize treatment strategies that reproducibly establish this immunological state instead of merely attempting to elicit sporadic abscopal responses.

## Conclusions

The abscopal effect represents a unique manifestation of systemic antitumor immunity elicited by local radiotherapy (10, 78). Although substantial progress has been made in elucidating its underlying mechanisms and developing rational radio-immunotherapy combinations, major challenges remain in patient selection, treatment optimization, toxicity management, and prospective clinical validation (111, 114, 129). Continued integration of tumor immunology, translational research, systems biology, and precision oncology will be essential to transform the abscopal effect from an unpredictable clinical observation into a reproducible therapeutic strategy (111). Achieving this goal has the potential not only to redefine the role of radiotherapy in systemic cancer treatment but also to establish a broader paradigm for the development of next-generation immune-based therapies.
